# Binding Interactions of Keratin-Based Hair Fiber Extract to Gold, Keratin, and BMP-2

**DOI:** 10.1371/journal.pone.0137233

**Published:** 2015-08-28

**Authors:** Roche C. de Guzman, Shanel M. Tsuda, Minh-Thi N. Ton, Xiao Zhang, Alan R. Esker, Mark E. Van Dyke

**Affiliations:** 1 School of Biomedical Engineering and Sciences, Virginia Polytechnic Institute and State University, Blacksburg, Virginia, United States of America; 2 Department of Chemistry, Virginia Polytechnic Institute and State University, Blacksburg, Virginia, United States of America; University of Quebect at Trois-Rivieres, CANADA

## Abstract

Hair-derived keratin biomaterials composed mostly of reduced keratin proteins (kerateines) have demonstrated their utility as carriers of biologics and drugs for tissue engineering. Electrostatic forces between negatively-charged keratins and biologic macromolecules allow for effective drug retention; attraction to positively-charged growth factors like bone morphogenetic protein 2 (BMP-2) has been used as a strategy for osteoinduction. In this study, the intermolecular surface and bulk interaction properties of kerateines were investigated. Thiol-rich kerateines were chemisorbed onto gold substrates to form an irreversible 2-nm rigid layer for surface plasmon resonance analysis. Kerateine-to-kerateine cohesion was observed in pH-neutral water with an equilibrium dissociation constant (K_D_) of 1.8 × 10^−4^ M, indicating that non-coulombic attractive forces (i.e. hydrophobic and van der Waals) were at work. The association of BMP-2 to kerateine was found to be greater (K_D_ = 1.1 × 10^−7^ M), within the range of specific binding. Addition of salts (phosphate-buffered saline; PBS) shortened the Debye length or the electrostatic field influence which weakened the kerateine-BMP-2 binding (K_D_ = 3.2 × 10^−5^ M). BMP-2 in bulk kerateine gels provided a limited release in PBS (~ 10% dissociation in 4 weeks), suggesting that electrostatic intermolecular attraction was significant to retain BMP-2 within the keratin matrix. Complete dissociation between kerateine and BMP-2 occurred when the PBS pH was lowered (to 4.5), below the keratin isoelectric point of 5.3. This phenomenon can be attributed to the protonation of keratin at a lower pH, leading to positive-positive repulsion. Therefore, the dynamics of kerateine-BMP-2 binding is highly dependent on pH and salt concentration, as well as on BMP-2 solubility at different pH and molarity. The study findings may contribute to our understanding of the release kinetics of drugs from keratin biomaterials and allow for the development of better, more clinically relevant BMP-2-conjugated systems for bone repair and regeneration.

## Introduction

**Fig 1:**
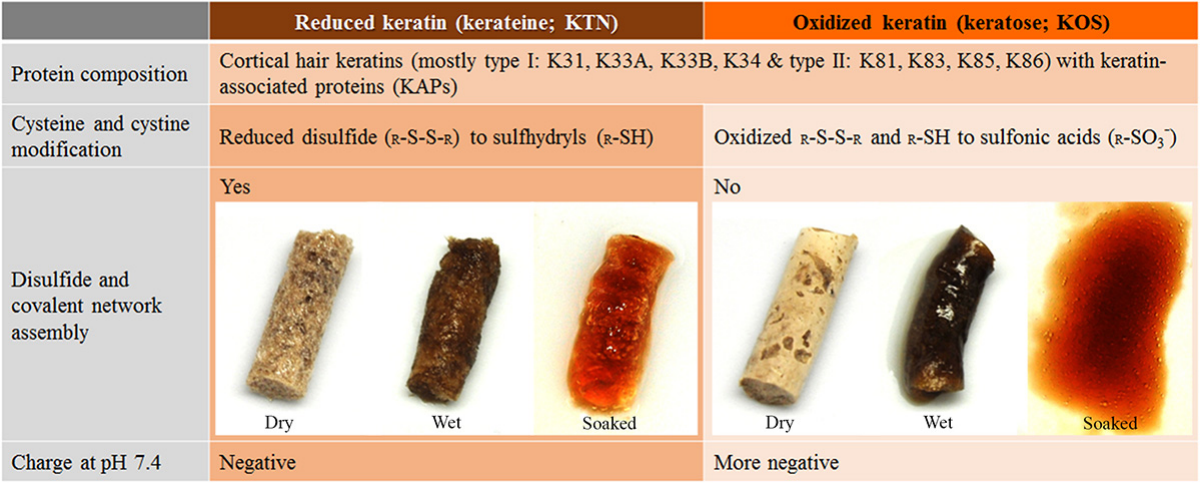
Comparison between reduced (KTN) and oxidized (KOS) keratin biomaterials. KTN can form disulfide linkages to produce a stable scaffold; but KOS cannot, due to the sulfonic acid modification of thiol groups. Consequently, the electrostatic properties are also altered, resulting in more negatively-charged KOS scaffolds, prone to more rapid hydrolytic degradation than KTN scaffolds. 10 mM NaOH solvent was used for wetting and soaking.

Keratins are members of the intermediate filament superfamily of cytoskeletal proteins that provide mechanical strength and support for cells.[1–4] Keratin-based extracts from the hair fiber cortex are now being used as extracellular matrix (ECM)-like biomaterials[5, 6] for a variety of tissue engineering applications, including local drug-delivery.[7, 8] The process to obtain working materials from hair keratins involves the reduction of the covalent disulfide bonds (R-S-S-R) of intra- and inter-molecular chain cystines (two linked cysteine amino acid residues) to form free thiols (R-SH), thus breaking the tough hair network and allowing the solubilization of keratin molecules. This reduced keratin product is called kerateine (KTN) and can eventually be oxidized to reform stable disulfide bonds. Alternatively, hair can initially be treated with peracetic acid to oxidize and modify cystines and cysteines to then generate cysteic acids containing negatively-charged sulfonic acid (R-SO_3_^-^) groups. Oxidized keratin extract is referred to as keratose (KOS) (Fig 1). Since KOS essentially lacks free thiols, its suprastructural network assembly only depends on non-covalent interactions; consequently, the bulk degradation of KOS is faster compared to KTN constructs.

Both KTN and KOS have been fabricated into films, gels, and scaffolds via suprastructural assembly of closely-packed protein subunits for use as carriers to retain and subsequently deliver bioactive drugs and growth factors.[7–17] The pharmacological release kinetics of these loaded compounds dictate the desired therapeutic effects and are influenced by the surface and bulk interaction of the drugs with keratins, as well as by the keratin degradation behavior. Hence, understanding the intermolecular interactions between keratins and the payload compounds will be helpful in designing better and more effective keratin-based implantable constructs and medical devices.

Previous results from our research group using KOS hydrogel scaffold as a carrier indicated that bone morphogenetic protein 2 (BMP-2), a clinically approved, highly potent growth factor that has been used to induce bone formation *in vivo*, has the potential to electrostatically bind to keratin.[11] In the case of KTN hydrogels, network structure arises from a combination of protein self-assembly and random entanglements, and is reinforced by disulfide bond formation, a feature that is lacking in KOS hydrogels. In either case, BMP-2 to keratin binding is hypothesized to be the result of the difference in net charge at physiological pH [at a pH of 7.4, the negatively charged KTN[18] with an acidic isoelectric point (pI = 5.3) is expected to associate with the positively charged BMP-2 (basic pI = 9)]. To better understand the underlying mechanisms, the KTN-to-KTN surface and bulk interactions at different pH and salt concentrations were also quantified. Moreover, globular proteins with acidic, neutral, and basic pIs were incorporated within KTN gels and the release profiles of these loaded proteins were determined to verify the charge-dependent keratin sequestration. Using surface plasmon resonance (SPR), the deposition and immobilization of KTN on gold were analyzed. Finally, the equilibrium dissociation constant (K_D_) values of KTN interactions at given conditions were obtained via SPR to comparatively assess the strength of paired non-specific binding.

## Materials and Methods

### Chemicals

Thioglycolic acid (TGA), Tris base, NaOH, peracetic acid (PAA), HCl, potassium chloride (KCl), hydrogen peroxide (H_2_O_2_), ammonium hydroxide, sulfuric acid (H_2_SO_4_), sodium dodecyl sulfate (SDS), and Tween 20 were purchased from Sigma-Aldrich (St. Louis, MO). HyClone phosphate-buffered saline (PBS; 6.7 mM phosphates), composed of 154 mM NaCl, 1.06 mM KH_2_PO_4_, and 5.6 mM Na_2_HPO_4_ at pH 7.4, was obtained from Thermo Fisher Scientific (Waltham, MA). Acidic PBS (pH 4.5) was made by adding drops of HCl. Deionized water at 18.2 MΩ·cm electrical resistivity was used for all solution preparation, dilution, extraction, and washing steps.

### Preparation of keratins

Kerateine (KTN) powder was prepared based on the modified protocol by Goddard and Michaelis.[19] Briefly, commercially-available human hair fibers (Chuchu's Hair Professional, New Orleans, LA) were reduced with 500 mM TGA, pH 11, at 50 g/L for 15 hr in a 37°C environmental shaker (100 rpm, mixing) and the resulting soluble proteins extracted.[20] The swollen hair mass was then subjected to additional 100 mM Tris base and water extractions, for 2 hr each. The TGA, Tris, and water extraction series was repeated, and extracts were pooled together, suspended solids separated by centrifugation and filtration to remove particles > 20-μm. The filtrate was dialyzed against endotoxin-free water using a 100-kDa membrane (Millipore, Billerica, MA) until the oxidation-reduction potential (ORP) of the system stabilized close to 0 mV (at 10–15 volumes to fully remove the excess TGA). The solution was concentrated to ~ 20 mg/mL, shell-frozen, and lyophilized. The dried KTN was ground using a blender, dispensed into vials, gamma ray-irradiated at 25 kGy, and stored, protected from moisture at -20°C. KTN samples were dissolved in 10 mM NaOH (pH 12) and concentrations were measured using a DC Protein Assay (Bio-Rad, Hercules, CA).

Oxidized keratin (KOS), lacking the thiol groups which were replaced by sulfonic acids, was utilized as a keratin control (Fig 1). KOS was processed according to the protocol by de Guzman *et al*.,[5] involving the treatment and oxidation of hair fibers with 2% PAA for 10 hr.

### Characterization of the KTN extract

The isoelectric point (pI) of keratins in the extract solution was empirically obtained by adding small, incremental amounts of HCl to the 20 mg/mL KTN (in 10 mM NaOH) solution while monitoring the pH. The recorded pI was the pH value when keratins started to precipitate in equilibrium. The pI of KOS was also measured.

Molecular weight (MW) of KTN components were determined using gel filtration chromatography (GFC). KTN powder was first solubilized in 50 mM KCl, pH 7.4 at 10 mg/mL. A 20-μL sample was then injected at 1 mL/min flow rate into the BioBasic SEC 300 column (300-Å pores; Thermo Fisher Scientific) with 50 mM KCl (pH 7.4) isocratic mobile phase. Proteins were detected at 280 nm using a Dionex UltiMate 3000 HPLC system (Thermo Fisher Scientific) UV detector, and absorbance versus retention time (RT) chromatograms were recorded using Chromeleon. Relative MWs of peaks were calculated using the equation:

$$MW\;=\;10^{\left(m*RT+b\right)}$$

where, MW = molecular weight (in kDa), RT = retention time (in min), m = slope, and b = y-intercept of the linear regression line of log(MW) versus RT of Gel Filtration Standard proteins (Bio-Rad) composed of thyroglobulin (670 kDa), γ-globulin (158 kDa), ovalbumin (44 kDa), myoglobin (17 kDa), and vitamin B_12_ (1.35 kDa). A separate KTN solution was acidified below the measured pI, spun at 4000 rpm for 10 min before the supernatant was collected and the precipitated pellet redissolved in 50 mM KCl, pH 12. The supernatant and solubilized pellet were adjusted to 10 mg/mL and pH to 7.4 and separately analyzed via GFC. Areas under the curve were quantified corresponding to the relative concentrations. The MW of KTN was also computed as the summation of the product of MW and fractional amount of individual components.

### Western blot of keratins

KTN and its “pellet” (keratin) and “supernatant” (keratin associated proteins) fractions were solubilized, neutralized, adjusted by mass (3 μg KTN), and separated by SDS polyacrylamide gel electrophoresis (SDS-PAGE) using a 4–20% gradient Mini-PROTEAN TGX gel (Bio-Rad) for 35 min at 200 V. BenchMark pre-stained protein ladder (Life Technologies, Grand Island, NY) was included as a control. Proteins were transferred onto a nitrocellulose membrane and blocked with 5% non-fat milk in Tris-buffered saline with 0.1% Tween 20 (TBS-T) for 2 hr. Guinea pig anti-human keratin-31 (K31) antibody (Progen Biotechnik, Heidelberg, Baden-Württemberg, Germany) at 1:2000 dilution in blocking buffer was used as a primary probe, then a 1:3000 dilution of rabbit anti-Guinea pig IgG-HRP (Life Technologies) was used as a secondary probe, each for a 1-hr incubation period. Membranes were washed three times in TBS-T after the primary and secondary hybridization steps. Pierce ECL Plus substrate (Thermo Fisher Scientific) mix was added for 2 min prior to chemiluminescent imaging with a ChemiDoc MP system (Bio-Rad).

### Adsorption of keratins on gold surface

Clean, gold-coated microscope slides (Au layer thickness ~ 50 nm; Fisher Scientific, Pittsburgh, PA) were soaked in 10 mg/mL KTN dissolved in 10 mM NaOH for 10 min. Unbound materials were removed by vigorously shaking the slides in water three times for 1 min each. The surface of the slides were dried using nitrogen gas. This procedure was repeated with clean Au-coated slides soaked in KTN solution overnight (~ 18 hr). Control groups were also made using 10 mM NaOH only (solvent control), and 10 mg/mL KOS in 10 mM NaOH (oxidized keratin control) treatments.

Surfaces (1 × 1 μm^2^ regions) were imaged using a BioScope II (Veeco, Plainview, NY) atomic force microscope (AFM) in tapping mode with a silicon cantilever (5–10 nm tip radius; 200–400 kHz resonance frequency). 3D surface plots with scaled-up z-axis and roughness measurements represented by root mean square (R_q_) values were obtained with the NanoScope.

To determine the elemental identities of the monolayer adsorbates on gold, X-ray photoelectron spectroscopy (XPS) was performed via the PHI Quantera XPS Scanning Microprobe system (Physical Electronics, Chanhassen, MN) with monochromatic Al K_α_ X-ray source (200-μm beam at a 45° take-off). Wide-scan spectra were recorded from 1100 to 0 eV and analyzed using Multipak. High-resolution binding energy regions for carbon (Cs1), sulfur (S2p), and gold (Au4f) were also evaluated at 0.1 eV step size for bonding and elemental states. Fityk 0.9.8 software[21] was then employed to remove Shirley and spline backgrounds.

### Surface plasmon resonance (SPR) analysis

KTN layer thickness and binding interactions were investigated using SPR. Plain gold surface sensor chips (12 mm × 12 mm square; Reichert, Buffalo, NY) were irradiated in a UV cleaner for 20 min, boiled in “base piranha” solution composed of 4% H_2_O_2_ and ammonium hydroxide (at 4% NH_3_) for 30 min, rinsed with water, and then placed in “piranha” solution (9% H_2_O_2_ and 70% H_2_SO_4_) for 30 min to oxidize and remove potential organic contaminants on the surface. After rinsing with water and drying, a chip (gold surface up) was placed onto the prism of the SR 7000 SPR Refractometer (Reichert) with a drop of immersion oil. The flow chamber cover with gasket was mounted and inlet and outlet tubing were connected. The machine was calibrated, a baseline was established, and the refractive index measurements were zeroed. All solutions were also degassed using a vacuum pump setup prior to the SPR run. Flow rate was set at 0.2 mL/min and the temperature was equilibrated to 20°C = 293.15 K. The 10 mM NaOH (solvent) was initially flowed until equilibrium was reached, followed by a 2.24 mg/mL KTN solution for 3 hr, solvent for 40 min, 2% SDS for 10 min, and a final solvent wash for 15 min. Surface concentration (Γ) of KTN on the gold surface was determined according to de Feijter’s equation:[22]

$$\Gamma =h_{A}\frac{n_{A}-n_{s}}{dn/dc}\;;$$

$$h_{A}=\frac{\Delta n}{dn/dh}\;;$$

$$\Gamma =\frac{\Delta n}{dn/dh}\cdot \frac{n_{A}-n_{s}}{dn/dc}$$

where, h_A_ = adsorbate layer thickness, n_A_ = refractive index of the adsorbate, n_s_ = refractive index of the solvent, dn/dc = refractive index increment of adsorbate in the solvent, Δn = SPR response (in μRIU), and dn/dh = 0.04°/nm = 6.83 × 10^11^ μRIU/m (machine constant, originally obtained[23] from Fresnel simulation). Differential refractometry using increasing concentrations of KTN in 10 mM NaOH (0, 0.25, 0.5, 1, and 2.24 mg/mL) and other analytes was done in Optilab (Wyatt Technology, Dernbach, Rhineland-Palatinate, Germany) to get the dn/dc constant. Using the computed Γ, KTN layer thickness (h_A_’) was determined to be:

$$h_{A}\prime =\frac{\Gamma }{\rho }$$

where, ρ = skeletal or absolute density of keratin[5] = 1.2 g/mL = 1.2 × 10^9^ mg/m^3^.

### Quartz crystal microbalance with dissipation monitoring (QCM-D) test

QCM-D was performed to infer the water content, as well as the rigidity of the adsorbed KTN layer. QSX301 gold-coated sensor chips (circle d = 14 mm; Q-Sense / Biolin Scientific, Gothenburg, Västra Götaland, Sweden) were cleaned as described for the SPR procedure, placed in flow cells, and mounted onto the E4 (Q-Sense) QCM-D work station. Water was flowed to establish a baseline before a 10 mM NaOH (solvent) was flowed for 10 min, followed by KTN solution at 2.24 mg/mL concentration for 2 hr, solvent for 20 min (to retain only the irreversible adlayer), and finally water for 10 min. The applied frequency was tuned to the resonant frequency (f_0_) of the quartz crystal (~ 5 MHz). Changes in frequency (Δf) per odd overtones (n), or Δf/n, and changes in dissipation parameter (ΔD) were simultaneously recorded. Values at n = 5 were selected for analysis. For ΔD < 1 × 10^−6^ per |Δf/n| = 10 Hz[24], Sauerbrey’s equation[25] was utilized to approximate the surface concentration (Γ’) of KTN, including any associated water:

$$\Gamma \prime =-C\frac{\Delta f}{n}$$

where, C = quartz crystal constant 0.177 Hz^-1^·mg/m^2^, for n = 1 and f_0_ = 5 MHz.

### KTN-to-KTN molecular interaction

Surface intermolecular interactions of KTN species were evaluated with SPR. The KTN extract, irreversibly bound monolayer (as ligand = B) was first deposited on gold as described above. KTN solution (as analyte = A) was then flowed for 15 min to induce KTN-KTN association and subsequently washed for 10 min with solvent (10 mM NaOH or water) for dissociation. KTN analytes were introduced at increasing order of concentrations: 62.5, 125, 250, 500, 1000, and 2240 μg/mL (0.6, 1.3, 2.6, 5.1, 10.2, and 22.9 μM using KTN MW = 98 kDa). Sensograms were plotted and overlaid at zero initial times and SPR responses. The Langmuir adsorption isotherm model was utilized to generate the following association-dissociation equations:[26–31]

Association phase:

$$R_{t}=R_{max}(1-e^{-k}{obs}^{t})\;;$$

$$R_{max}=B_{max}(occupancy)\;;$$

$$occupancy\;=\;\frac{\left[A\right]}{K_{D}+[A]}\;;$$

$$K_{D}\;=\;\frac{k_{d}}{k_{a}}\;;$$

$$k_{obs}\;=\;k_{a}\left[A\right]+k_{d}\;;$$

$$R_{t}=B_{max}\left(\frac{\left[A\right]}{\frac{k_{d}}{k_{a}}+\left[A\right]}\right)\;\left(1-e^{-(k_{a}\left[A\right]+k_{d})t}\right)$$

where R_t_ = Δn = SPR response (in μRIU), R_max_ = maximum SPR response, k_obs_ = observed rate constant, t = time (in s), B_max_ = maximum ligand binding response, [A] = concentration of analyte (in M), K_D_ = equilibrium dissociation constant, k_d_ = dissociation rate constant (in s^-1^), and k_a_ = association rate constant (in M^-1^s^-1^).

Dissociation phase:

$$R_{t}=R_{0}(e^{-k_{d}t})$$

where, R_t_ = SPR response (in μRIU), R_0_ = SPR response at time 0 (start of dissociation), k_d_ = dissociation rate constant (in s^-1^), and t = time (in s). B_max_, k_a_, and k_d_ constants were obtained using the Office Excel (Microsoft, Redmond, WA) solver tool by minimizing the deviation of the expected R_t_ from the observed with least squares regression. Prism (GraphPad, San Diego, CA) was then employed to fit a line to the XY scatter plot of x = [A] and y = k_obs_, with the slope (m) = k_a_, and the y-intercept (b) = k_d_. The computed K_D_ value (= k_d_/k_a_) was converted to dissociation energy (ΔG) using:

$$\Delta G=-RT\cdot ln\frac{{\text{K}}_{\text{D}}}{c^{ɵ}}$$

where R = ideal gas constant 1.987 × 10^−3^ kcal·K^-1^mol^-1^, T = temperature 293.15 K, and c^ɵ^ = standard reference concentration 1 M. The half-life (t_½_) was also reported as:

$$t_{½}=\frac{\text{ln}(2)}{k_{d}}\;.$$

### KTN-to-KTN bulk interaction

Insights on bulk KTN-KTN interactions were obtained with the gel degradation assay. Under sterile conditions, 10% (100 mg/mL) KTN gels were made using solvents at varying pH (2, 5, 7.4, 10, and 12) and KCl concentration (0, 75, 154, 300 mM), then immediately placed into wells of a 96-well microplate (120 μL each), centrifuged and allowed to stabilize overnight (thiol oxidation to form disulfide crosslinks). Gels were washed three times with their corresponding solvents, before 120 μL of solvent (supernatant) was placed on top of the gels, and the gels incubated in a sealed container at 37°C. Supernatants were collected and replaced with fresh solvent at 0, 0.125, 0.25, 0.5, 1, 2, 4, 7, 14, and 28 d. KTN protein mass of the supernatants were measured and converted to degradation (%) by dividing by the original gel mass of 12 mg. Cumulative degradation values were plotted versus time.

### Bone morphogenetic protein 2 (BMP-2)-to-KTN interaction

SPR flow experiments were conducted to determine the interaction of BMP-2 (as analyte = A) with immobilized KTN (as ligand = B) on gold. Recombinant CHO mammalian cell-expressed human BMP-2 (pI = 9; Medtronic, Minneapolis, MN)[32] was dissolved in the following solvents: PBS at pH 7.4, PBS at pH 4.5, and water, before they were serially-diluted to 250, 125, 63, 31, 16, 8, 4, 2, and 1 μg/mL (6944, 3472, 1736, 868, 434, 217, 109, 54, and 27 nM using dimeric BMP-2 MW = 36 kDa[33, 34]). BMP-2 analytes were flowed over immobilized KTN substrates starting from the lowest to the highest concentration. After each association step, bound molecules were dissociated with their respective solvents followed by regeneration with 2% SDS and solvent washes to completely remove any residual BMP-2.

### Release of proteins from bulk KTN gels

100 mg/mL KTN gels were casted (to make an 800:1 mass ratio of KTN to loaded protein) with one of the following proteins at 125 μg/mL: bovine serum albumin (pI ~ 5, MW = 66 kDa), human hemoglobin (pI ~ 7, MW = 64 kDa), and chicken egg white lysozyme (pI ~ 11, MW = 14 kDa)[35–37] (all purchased from Sigma-Aldrich), dissolved in 50 mM KCl, pH 7.4 (solvent). Equal volumes of solvent were added on top of the gels before placing in a 37°C incubator. At 0, 1.5, 3, 6, 12, and 24-hr time points, supernatants were collected and replaced with fresh solvents. The 0, 6, 12, and 24-hr samples were evaluated via GFC on an SEC 300 column. Peaks corresponding to albumin, hemoglobin, and lysozyme were selected and concentrations were determined by measuring the areas under the curve.

BMP-2 release out of bulk KTN was also monitored. 80 mg/mL of KTN was thoroughly mixed with 100 μg/mL BMP-2 (800:1 mass ratio) in PBS (pH 7.4), centrifuged, and allowed to form a stable gel overnight. KTN-BMP-2 gels were placed in a 10-fold volume of PBS at 37°C. Aliquots of the fluid were obtained at 0, 0.0625, 0.125, 0.25, 0.5, 1, 2, 4, 7, 14, 21, and 28 d and replaced by the same volume of fresh PBS. The collected products were evaluated with a DC Protein Assay (total proteins) and with a BMP-2 enzyme-linked immunosorbent assay (ELISA) kit (PeproTech, Rocky Hill, NJ). The amount of BMP-2 was subtracted from the total protein concentration to obtain the KTN extract concentration. The BMP-2 release profile was normalized to the KTN only background ELISA signal.

### Keratin extract scaffolds

10% KTN and 20% KOS in 10 mM NaOH solvent were separately injected into the lumen of a Nalgene 890 polytetrafluoroethylene tubing (id = 4.8 mm; Nalge Nunc, Rochester, NY) at 200 μL each (length ~ 11 mm). After overnight incubation, gels were frozen, lyophilized, and removed from the tubing (dry). 200 μL of solvent was added to the dry scaffolds (wet). After 30 min, the scaffolds were then immersed in the solvent overnight (soaked). Images were taken at their dry, wet, and soaked stages (Fig 1).

### Data management and statistical methods

Replicates were made with a sample size of (n) ≥ 3. Computed values and bar graph representations were reported as mean ± 1 standard deviation. Representative curves were generated using Excel and Prism. Figures were drawn and processed via PowerPoint (Microsoft) and Photoshop (Adobe, San Jose, CA). Linear regression, Student’s t-test, and one-way and two-way analysis of variance (ANOVA) combined with post-hoc multiple comparison tests (Tukey’s and Bonferroni correction, respectively) were performed with Prism at 95% confidence intervals and probability of type I error (α) at 0.05.

## Results and Discussion

### Kerateine properties

The reduced keratin (KTN) extract is mostly composed of cortical hair keratin proteins: K31, K33A, K33B, K34, K81, K83, K85, and K86 (Fig 1).[20] The relative amounts of each protein are still undetermined, but assuming type I (K31-34) and type II (K81-86) keratins occur in equimolar ratios, the ExPASy Proteomics server[38] and Universal Protein Knowledgebase (UniProtKB)[39] predicted average isoelectric point (pI) of KTN is 5.3 ± 0.5 and the molecular weight (MW) is 51 ± 4 kDa. Precipitation is a method of determining the protein’s pI since the zero net charge leads to decreased water interaction and solubility.[40, 41] Using this approach, the pI of KTN was found to be 5.3 ± 0.1 (n = 5), matching the expected pI. At pH ≤ pI, keratin proteins aggregated and precipitated out of solution. Readjustment to pH 6 solubilized all precipitates. This precipitation and solubilization phenomenon was observed for 5 cycles, suggesting reaction reversibility. By comparison, the obtained pI of the oxidized hair keratin extract (KOS) = 4.2 ± 0.1 (n = 4). Oxidation and modification of cysteines and cystines to cysteic acids lowered (acidified) the overall pI of protein constituents,[42, 43] which also resulted in the accumulation of more negative charges to KOS.

**Fig 2:**
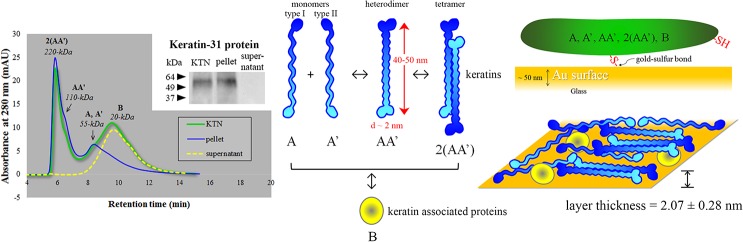
KTN characterization. The KTN extract is comprised of monomeric (A, A’), dimeric (AA’), and tetrameric (2(AA’)) keratins as well as keratin associated proteins (B) indicated by peaks corresponding to their respective molecular weights using gel filtration chromatography. One of the protein monomers detected by western blot technique is keratin-31 (K31). Both keratins and keratin associated proteins have free thiols (R-SH) that react with gold and form strong gold-sulfur bonds. Au-S covalent interactions, as well as some physisorption, enable the formation of a stable KTN monolayer.

Gel filtration chromatography (GFC) generated the equation: MW = 10^(-0.2639*RT+3.946)^ for the protein standards. MW values were then computed from retention time (RT) peaks. The KTN solution showed two major peaks at ~ 220- and 20-kDa (Fig 2, green curve). To investigate further, KTN was fractionated into a pellet and a supernatant after lowering the pH to less than the measured pI (< 5.3). GFC of neutralized components revealed that the “pellet” was comprised of keratins at their monomeric (55 kDa), heterodimeric (110 kDa), and tetrameric (220 kDa) forms, distributed at a ratio of 38%, 14%, and 48%, respectively (Fig 2, blue curve); while the “supernatant” represented keratin associated proteins with an average MW of 20 kDa (Fig 2, yellow dashed curve). The bimodal shape and relative abundance of keratin monomers and tetramers indicate that the dimers act as intermediates between the dynamic conversion of monomers to tetramers and vice versa (Fig 2, middle diagram). Furthermore, quantification of areas under the curve suggested that the KTN extract was made up of 64% keratins and 36% keratin associated proteins. The low-MW keratin associated proteins have a predicted pI ranging from 8–9, hence remain in solution at acidic pH.[20, 38] The recorded 55-kDa keratin monomer MW is higher than the 51-kDa predicted value. This discrepancy can be accounted for by post-translational modifications like glycosylation, acetylation, and phosphorylation.[44] Interpreted as a single unit, the KTN extract mean MW was calculated to be: ((55 kDa)(0.38) + (110 kDa)(0.14) + (220 kDa)(0.48))(0.64) + (20 kDa)(0.36) = 98 kDa.

Western blotting confirmed that one of the major intermediate filament proteins of hair cortex, keratin 31 (K31), was indeed present in the KTN extract (Fig 2, inset). The observed MW was ~ 55 kDa, slightly higher than the predicted 47 kDa, but supported the data obtained from the GFC size calculation of monomeric keratins. K31 was localized to the pellet fraction and was not found in the supernatant, indicating that the 20-kDa protein GFC peak did not correspond to a keratin protein.

### Surface interactions between keratins and gold

**Fig 3:**
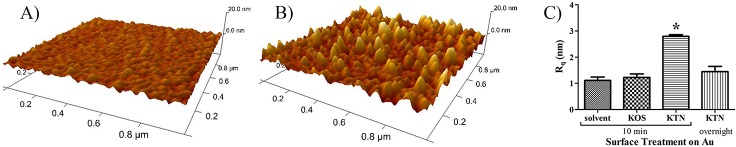
Layer roughness. Gold surfaces treated with A) solvent only (10 mM NaOH) and with KOS (oxidized keratin) solution have relatively smooth profiles compared to those with B) KTN (reduced keratin) extract. C) Partial surface adsorption of KTN increased the roughness, Rq, whereas full coverage by overnight KTN incubation led back to a smoother surface. *p < 0.05 compared to each of the other groups.

Static incubation of a 10 mg/mL KTN for 10 min on gold resulted in a rougher surface compared to the solvent-only treatment (Fig 3), indicative of KTN adsorption on Au. The obtained root mean square roughness (R_q_) values were as follows: 1.11 nm, 1.23 nm, 2.80 nm, and 1.45 nm for solvent, KOS, KTN, and KTN-overnight treatments, respectively. Analysis of variance (ANOVA) demonstrated significant differences in means (p = 0.0009) and Tukey’s comparison showed that the KTN-10 min group had a higher R_q_ (p < 0.05) than the others. The R_q_ of the partially-covered gold surface with KTN can be used to approximate the thickness of the KTN layer. Prolonged KTN incubation (overnight) led to a smoother surface topography, indicating complete KTN adsorption, and minimization of the Au solid surface-associated energy.

**Fig 4:**
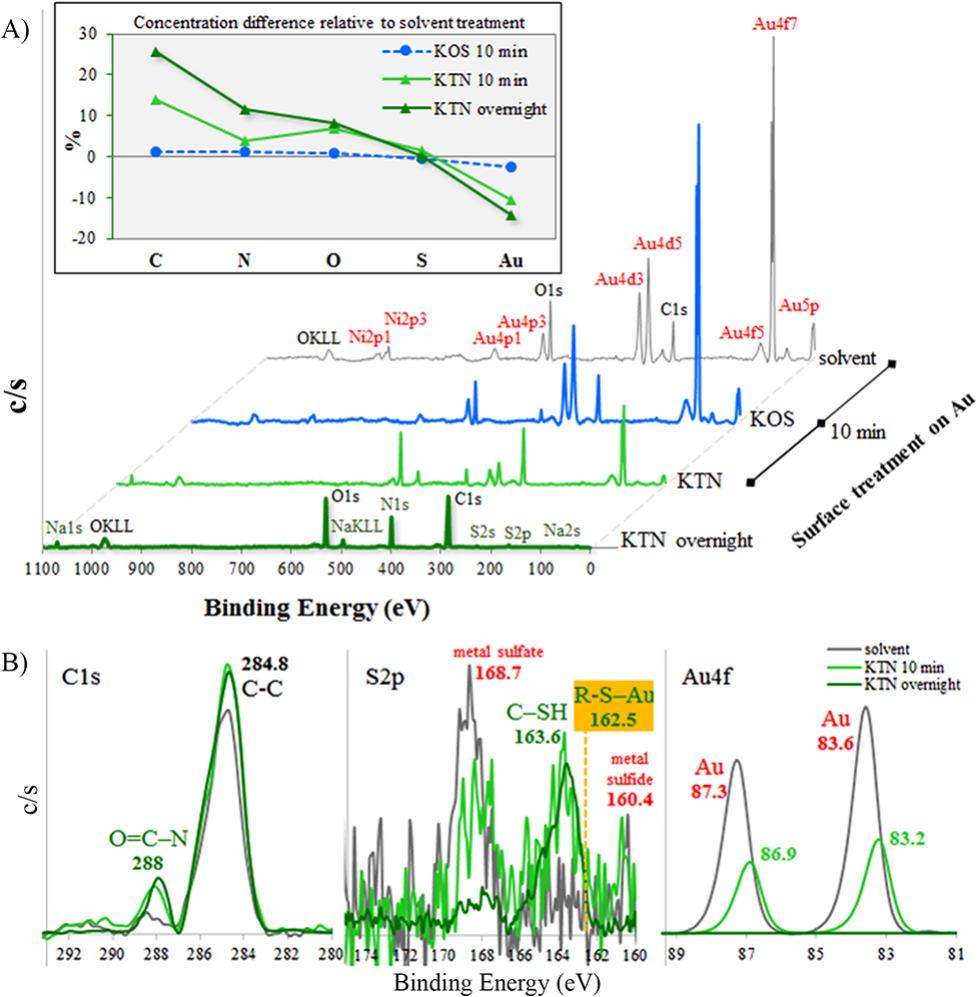
XPS analysis. A) Wide-scan XPS spectra of gold surfaces treated with 10 mM NaOH solvent, KOS, and KTN. Overnight incubation of KTN led to no detectable Au signals. The inset graph shows that, compared to the solvent group, KOS has very similar concentration levels of carbon, nitrogen, oxygen, sulfur, and gold, while KTN has elevated amounts of protein elements (C, N, and O) but decreased Au. B) Near-scan analysis displays the formation of an amide (O = C-N) peak at 288 eV, corresponding to KTN protein deposition on gold. Unbound and gold-bound KTN thiols were also detected at 163.6 and 162.5 eV, respectively. Partial adsorption of KTN on gold shifted the Au4f peaks to slightly lower energies.

Wide-scan X-ray photoelectron spectroscopy (XPS) data revealed that the solvent-treated control group (bare gold surface) corresponded to a depth of up to about 10 nm[45] and contained 35.2% C, 3.2% N, 11.4% O, 0.7% S, and 14.2% Au among the 13 detected elements (C, N, O, F, Na, Mg, Si, S, Cl, Ca, Ni, Zn, and Au). Compared to the uncoated control, the KOS coated gold had 1.2% more C, 1.3% more N, 0.7% more O, 0.4% less S, and 2.5% less Au (Fig 4A), indicating very little adsorption and interaction to gold. Conversely, KTN demonstrated a greater ability to adsorb on gold, and KTN-treated groups showed a significant increase in protein elements: 13.9% C, 3.8% N, 7.0% O, and 1.4% S, and decrease in Au (at 10.7% less). Overnight incubation of KTN produced no detectable Au signal, signifying complete coverage of proteins on the gold surface as the KTN layer depth was beyond the reach of the X-ray beam to eject any Au electrons. This adsorbed KTN had a relative composition of about 61% C, 15% N, and 20% O, closely resembling the thick albumin protein film values[46] (~ 65% C, 14% N, and 18% O).

An amide (O = C-N) peak[47, 48] of ~ 288 eV binding energy was observed in high-resolution XPS for KTN treated groups (Fig 4B), which confirmed the adsorption of amino acids on the gold surface. In the S2p region, a 163.6-eV peak was recorded for KTN groups, representing the free thiols (C-SH)[49] in KTN species. The 162.5 eV signal for gold-bound thiol (R-S-Au)[50–52] was partially buried in the free thiol curve. The bare gold surface generated two expected peaks at 87.3 and 83.6 eV for Au electron signals in the 4f5 and 4f7 orbitals, respectively.[53] Partial KTN adsorption lowered the intensities and shifted these peaks to 86.9 and 83.2 eV, but these signals can still be attributed to the presence of metallic gold.[54]

Coating with the KOS control extract (lacking any free thiols) resulted in very small amounts of adsorption or physisorption (van der Waals interaction) on gold surfaces. Therefore, atomic force microscopy (AFM) and XPS results suggest that KTN deposition is mainly due to chemisorption, specifically via free thiol interactions with gold to create sulfur-gold covalent bonds[55] (Fig 2). Despite the chemisorption process, an excess of unbound thiols was still present in the immobilized KTN groups, allowing for other interactions.

The strong binding-association of sulfur (in thiolated proteins like KTN) to gold [56] is also observed in other metals including silver.[57] In fact, metallothioneins (proteins rich in thiol groups) are known to bind to a variety of metallic species (such as Au, Ag, and Cu) in the physiological and xenobiotic environment.[58] Researchers have utilized this thiol-metal affinity approach to conjugate proteins on metal nanoparticulates.[59–61]

### Keratin adlayer thickness

**Fig 5:**
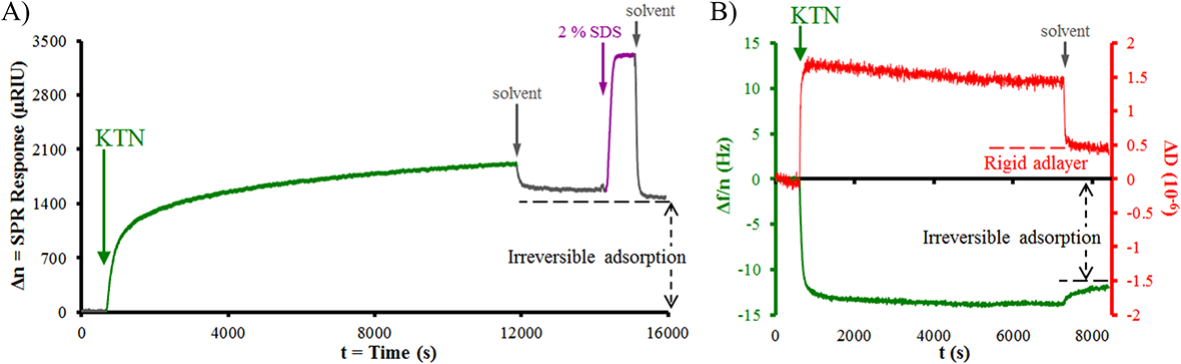
Interface behavior. Adsorption kinetics of KTN on gold analyzed through A) SPR and B) QCM-D methods. After establishing baseline readings using 10 mM NaOH solvent, KTN solution was flowed on gold sensor chips until almost full saturation. A) The irreversible adlayer at SPR response (Δn) = 1410 μRIU was retained after solvent, 2% SDS, and solvent washings, and its surface concentration (Γ) = 2.49 mg/m2. B) The normalized changes in dissipation factor (ΔD) for the adsorbate = 0.46 × 10–6 (red curve, using the right y-axis), suggest rigidity. The changes in frequency per overtone (Δf/n) of the adsorbed KTN = -12.08 Hz (green curve, using the left y-axis), correspond to a surface concentration (Γ’) = 2.14 mg/m2. A comparison between Γ (SPR) and Γ’ (QCM-D) showed statistical similarity (p = 0.4273).

Surface plasmon resonance (SPR) sensogram of the KTN adsorption kinetics displayed a very sharp rise during the first 20 min of flow (indicative of high-affinity binding), followed by a pseudoplateau (Fig 5A) similar to polymer irreversible adsorption behavior which can be modeled by the Freundlich adsorption isotherm.[27, 62] As the surface gets occupied and filled up by the adsorbate, it becomes increasingly harder to allow more adsorption; thus, saturation and equilibrium will never be reached (i.e. pseudo-saturation). A true plateau was established after the removal of the lightly-adhered, 1.38-nm reversibly-bound KTN layer with the washing solvents. Tightly-bound KTN remained associated with the gold surface post-treatment with 2% sodium dodecyl sulfate (SDS) detergent solution. Differential refractometry was utilized to determine the following optical properties: the dn/dc of KTN in a given solvent = 0.1595 mL/g, the refractive index of KTN adsorbate (n_A_) = 1.5276, and the refractive index of 10 mM NaOH solvent (n_s_) = 1.3331. These, together with the dn/dh machine constant and the SPR response (Δn = R_t_) in the irreversible KTN adsorption region, were employed to solve for the maximum or saturation surface concentration (Γ), which was found to be 2.49 ± 0.34 mg/m^2^ (n = 4). From these values, the KTN adsorbate thickness (h_A_’) was approximated = 2.07 ± 0.28 nm, statistically-similar to the value obtained using AFM on gold samples with partial KTN adsorption (2.8 nm; p = 0.2966). Intermediate filament protein dimers (including keratins), have 40–50 nm rod lengths and an average diameter of 2 nm,[63] suggesting that the adsorbate was mostly a monolayer of “train” keratins or the conformation of rod-shaped keratins was “side-on” with the long axis parallel to the surface[64] (Fig 2). The abundant tetrameric keratin form was likely oriented so that both dimeric components adhered side-on to the gold surface, preserving the layer thickness of ~ 2 nm.

The changes in the dissipation factor (ΔD) per 10 Hz were detected as (0.46 ± 0.45) × 10^−6^ (n = 4) in the irreversible KTN adsorption region via quartz crystal microbalance with dissipation (QCM-D) analysis (Fig 5B, red curve). Since ΔD is < 1 × 10^−6^, the monolayer adsorbate is considered stiff and rigid.[24, 65] The low ΔD value corresponds to negligible energy loss and minimal damping of the quartz oscillation, making Sauerbrey’s equation valid.[24, 25] The computed KTN surface concentration, including any possible associated solvent molecules, (Γ’) based on the change in frequency per overtone (Δf/n) was calculated to be 2.14 ± 0.75 mg/m^2^ (n = 4) (Fig 5B, green curve). A student’s t-test comparison between Γ (SPR) and Γ’ (QCM-D) resulted in no statistical difference (p = 0.4273); therefore there was apparently no water association with the adlayer or any adsorbate layers.

### KTN intermolecular interaction

Association and dissociation analyses of ligand-analyte interactions were based on the assumption that the system followed a Langmuir isotherm model in which an analyte (A) non-covalently binds to the surface-immobilized ligand (B) in a 1:1 ratio to produce a fully reversible ligand-analyte complex (AB).[26–30] The strength of binding interaction was indirectly quantified by determining the equilibrium dissociation constant (K_D_) or the analyte concentration needed to saturate half of the available ligand sites. Additionally, the K_D_ value was translated to dissociation energy (ΔG) to compare relative bond dissociation energies of molecules. The half-life (t_1/2_) or the amount of time required to decrease the ligand-analyte-complex concentration by half was also reported. The lower the K_D_ and the higher the ΔG and t_1/2_, the stronger the analyte-to-ligand association.[66] One of the strongest known non-covalent biological compound interactions is biotin-to-avidin/streptavidin, with a K_D_ of ~ 10^−15^ to 10^−13^ M.[67–70] A highly-specific and strong antigen-antibody binding typically has a K_D_ ranging from 10^−11^ to 10^−9^ M.[71, 72] Cell adhesion via integrin receptors to extracellular matrix proteins like collagen, laminin, and fibronectin is characterized by a K_D_ of 10^−9^ to 10^−7^ M.[73–75] These low-K_D_ specific interactions are largely electrostatic in nature.[76] K_D_ values approximately 10^−6^ to 10^−5^ M are considered moderate, while ≥ 10^−4^ M corresponds to low-affinity binding, indicative of hydrophobic and van der Waals non-specific interactions.[76, 77]

**Fig 6:**
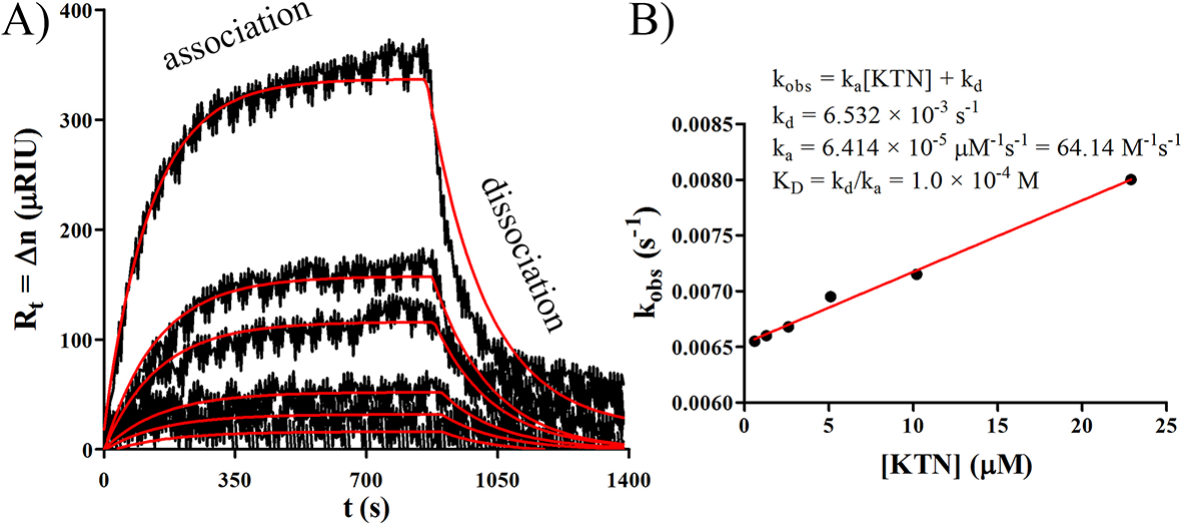
SPR interaction. A) Representative SPR sensogram of KTN-KTN association and dissociation with 10 mM NaOH, generated by increasing concentrations of the analyte (0.6, 1.3, 2.6, 5.1, 10.2, and 22.9 μM) and fitted with Langmuir curves (red trace). B) Linear regression plot of kobs vs [KTN] was used to obtain the slope = ka, y-intercept = kd, and KD = kd/ka.

KTN on KTN SPR experiments determined that the K_D_ for KTN in 10 mM NaOH (pH 12) = 1.0 × 10^−4^ M (corresponding to ΔG = 5.4 kcal/mol) and the t_1/2_ = 106 s (Fig 6), similar in K_D_ magnitude to the binding of albumin to small molecules.[78, 79] Because of the relatively high K_D_ and low t_1/2_, KTN intermolecular associations were possibly dominated by non-specific interactions of hydrophobic stripes on the surface of coiled-coil keratin rod domains[80] (hydrophobic interaction); these interactions are much weaker than the high-affinity and very specific association of type I and type II keratins that occurs during heterodimer formation [63, 81, 82].

Protein-protein interactions are dependent on temperature, pH, and salt concentrations.[83–85] Changing the solvent to water (pH 7) led to a slight increase in the K_D_ value to 1.8 × 10^−4^ M (ΔG = 5.0 kcal/mol) and a decrease in the t_1/2_ to 77 s. Phosphate-buffered saline (PBS), commonly used in biological-simulated environments due to similarities in pH and osmolarity with tissue fluids, was not used as a solvent because KTN was insoluble in PBS at the concentrations used for SPR analysis. Qualitative observations demonstrated that the higher the pH (more basic) and the lower the salt content of the solvent, the higher the KTN solubility. However, too much alkalinity can potentially lead to protein hydrolysis. Taking these into account, a good starting solvent for KTN was determined to be 10 mM NaOH (pH = 12).

**Fig 7:**
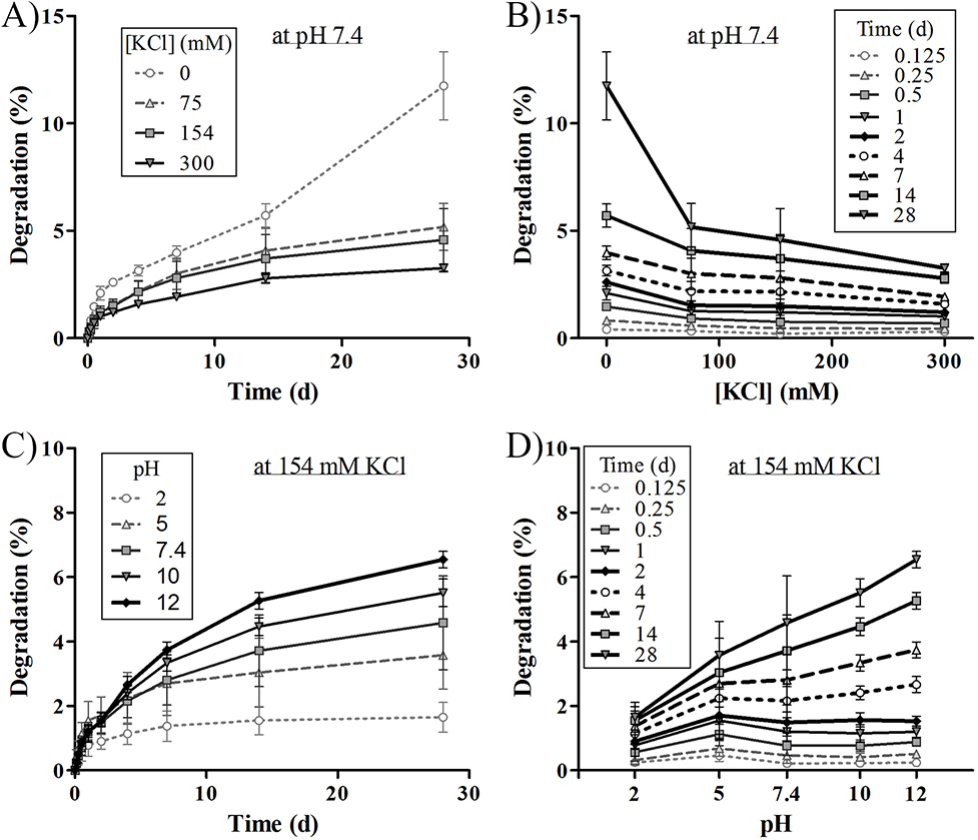
KTN bulk release. KTN gel bulk degradation in vitro at A-B) constant pH = 7.4, and C-D) constant [KCl] = 154 mM for over a period of 28 days at 37°C. Faster degradation occurred at longer time points, lower [KCl], and higher pH levels.

Total protein release from KTN gels was conducted to determine the KTN-KTN interaction behavior in bulk at varying time points, pH, and salt concentrations (represented by KCl). A solvent with 154 mM KCl at pH 7.4 was used to correspond to physiological medium. Slower degradation kinetics indicated stronger KTN intermolecular association. Analysis of 200 data points (10 time points × 5 pH levels × 4 [KCl]) revealed a general pattern: shorter times, higher acidity (lower pH), and higher KCl amounts translated to more stable gels (Fig 7 for results of samples containing 154 mM KCl and pH 7.4). Lower solubility conditions generated longer lasting gels. At pH 2, regardless of [KCl], gels were very stable; they degraded to only ~ 2% of their original mass after the 28-day time point. Protein release into the medium was more pronounced for KTN gels with no KCl salts added; at the end of the study, about 11%, 12%, 25%, and 37% degradation were observed for pH 5, 7.4, 10, and 12, respectively. In the absence of salts in the liquid layer, electrostatic or coulombic interactions dominate.[27, 86] The Debye length (net effective range of electrostatic field) is extensive in pure water (~ 950 nm). Addition of even just a few mM of KCl drastically reduces the Debye length,[87] which in turn increases the influence of van der Waals forces of interaction (and other non-electrostatic forces). At physiological salt level, the Debye length goes down to less than a nanometer. The fast degradation behavior of gels in zero KCl can therefore be mostly attributed to long-range repulsion of negatively-charged keratin[18] molecules. Gels with at least 75 mM KCl generated a maximum degradation of only 7%, since the Debye length decreased and consequently, coulombic effects were no longer significant. Increase in pH also leads to longer Debye length[86, 88] and greater negative-negative repulsive force; hence, the quickest bulk KTN protein release occurred in alkaline conditions. Conversely, lower pH shortens the Debye length and approaches the KTN pI of 5.3, bringing the net charge to neutrality, making water solvation harder, and promoting protein-protein aggregation due to hydrophobic amino acid interaction (nonpolar-nonpolar) and van der Waals attraction. At a pH less than the pI, keratins are positively-charged; but because of the greatly decreased Debye length and dominance of non-coulombic interactions, proteins still associate together. As the empirical data showed, low pH and higher salt concentration enabled stronger protein cohesion[89] and bundling of keratin filaments[82] resulting in more stable gels. It should be noted that unlike globular protein gels,[90, 91] fibrous KTN hydrogels formed under low pH and high salt content have a translucent appearance and are still able to absorb an excess of water.

The stability of KTN gels is also influenced by the covalent network of intermolecular disulfide bonds–more disulfides and stronger crosslinks result in less bulk degradation. In a living cell environment, disulfide bond formation between cysteines is mediated by enzymatic thiol/disulfide exchange reactions.[92–95] However, for the simple system of reduced keratin protein extract dissolved in aqueous media, the gelation mechanism and building of disulfide bridges occur in two ways: 1) air (oxygen) oxidation of thiols, releasing water in the process, and 2) direct thiol-thiol interaction which generates hydrogen gas as a byproduct.[96] Reaction 1 is a slow process that is enhanced under acidic pH;[89, 97] reaction 2 is relatively fast and happens only at high alkalinity. Gels were incubated overnight to ensure completeness of reaction. More stable KTN gels were formed at lower pH since disulfide bonds are preserved in acid but degraded at alkaline pH.[98] High salt content induces protein aggregation, bringing thiols in closer proximity to each other, promoting disulfide bond formation;[89] hence, less gel degradation was observed. For these stronger gels, it is expected that the non-covalently-linked KTN constituents will first be released into the medium prior to the disulfide-linked ones during KTN gel degradation.

### KTN-bone morphogenetic protein 2 (BMP-2) interaction

The surface interaction between KTN and BMP-2 was evaluated by SPR association-dissociation experiments. BMP-2 is highly soluble in water. Using very low BMP-2 concentrations (up to 434 nM), the KTN-to-BMP-2 K_D_ in water was found to be 1.1 × 10^−7^ M (ΔG = 9.3 kcal/mol) and the t_1/2_ = 83 min, which is suggestive of a strong specific binding interaction. The highest SPR signal response was exhibited at a critical concentration of 434-nM BMP-2, with a plateau occurring at 6312 μRIU, translating to Γ (surface concentration) = 11.09 mg/m^2^ and h_A_’ (thickness) = 9.2 nm (assuming full saturation) after employing the calculated constants: BMP-2 n_A_ = 2.3324, dn/dc = 0.8328 mL/g, and ρ = 1.2 g/mL. Human BMP-2 dimer has dimensions of 7 nm × 3.5 nm × 3 nm,[99] with a diameter = 5.2 nm in spherical form. Thus, approximately two (9.2 nm/5.2 nm) molecular layers of dimeric BMP-2 electrostatically adhered onto the KTN substrate interface. The equivalent concentration of BMP-2 in the fixed flow chamber with volume of 2.5 mm^3^ and surface area of 12 mm^2^ is 53 μg/mL = 1479 nM, while KTN = 122 nM. Accordingly, it seems that the optimal and most stable KTN:dimeric BMP-2 mole ratio is about 1:12.

**Fig 8:**
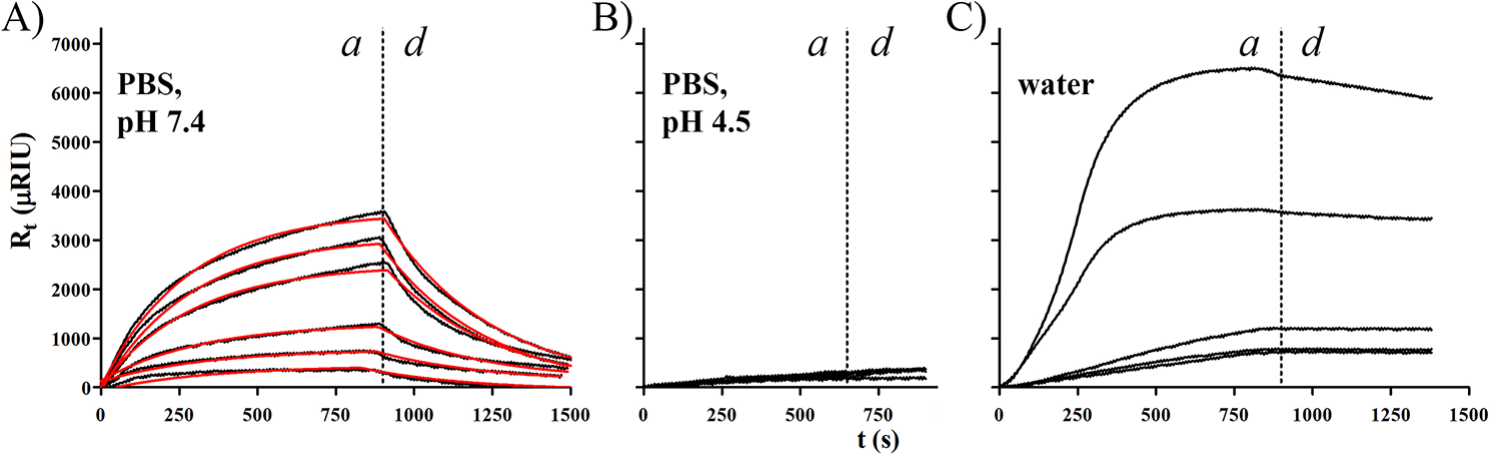
KTN-BMP-2 interaction. Association (a) and dissociation (d) electrostatic interaction profiles of BMP-2 and the KTN monolayer in PBS and in water. BMP-2 analytes were successively flowed, at incrementally increasing concentrations: A) in PBS (pH 7.4) at 0.2, 0.4, 0.9, 1.7, 3.5, and 6.9 μM, B) in PBS (pH 4.5) at 0.03, 0.05, 0.1, 0.2, 0.4, 0.9, and 1.7 μM, and C) in water (pH 7) at 0.03, 0.05, 0.1, 0.2, and 0.4 μM. KTN-BMP-2 electrostatic attraction was strongest in water (KD = 1.1 × 10–7 M). In the presence of PBS salts at physiological pH, binding association was slightly weakened (KD = 3.2 × 10–5 M). Acidification of the PBS eliminated any binding between BMP-2 and KTN.

Higher concentrations of BMP-2 (868 to 6944 nM in water) caused strong repulsive forces among free BMP-2 analytes, leading to lower binding affinity to KTN at higher BMP-2 concentrations (inverse response) and faster peak formation. This was also associated with quick, spontaneous complex detachment even in the association phase, making the Langmuir curves unusable. Ideal Langmuir association behavior is characterized by a rectangular hyperbolic curve (Fig 6A). However, BMP-2 binding to KTN in water generated sigmoidal curves (Fig 8C), indicating a non-1:1 association and mass transport limitation (MTL) behavior,[100] possibly due to steric hindrance and intermolecular coulombic repulsion among positively charged BMP-2 molecules. Increasing the flow rate or using linker chains to bind KTN ligands to gold may address this MTL issue.[30]

BMP-2 has limited solubility in PBS at pH 7.4 –it forms a clear solution at ≤ 1736 nM and a cloudy suspension at ≥ 3472 nM. Despite this, SPR data of KTN-BMP-2 interactions resulted in fittable regression curves (Fig 8A) that yielded a K_D_ value of 3.2 × 10^−5^ M (ΔG = 6.0 kcal/mol) and t_1/2_ of 224 s. The presence of PBS decreased electrostatic repulsion between ions, however the salt concentration still allowed moderately strong KTN-BMP-2 binding. At 434 nM, the plateau (R_t_ = 584 μRIU) represented a Γ of 1.02 mg/m^2^ and effective volume concentration of 137 nM, which is approximately a 1:1 KTN-BMP-2 binding (versus 1:12 ratio in water). Physiological salts in the medium thus diminished the intermolecular electrostatic interactions ~12-fold. The weakly negative attractive force of KTN attracted a 0.97-nm thick PBS layer, likely composed of Na^+^ and K^+^ cations, which together (with negatively charged KTN) formed an electric double layer (i.e. a Stern layer).[27] QCM-D data yielded a ΔD value (at 10 Hz) = 1.53 × 10^−6^, indicating that the Stern layer was viscoelastic (for ΔD > 10^−6^), which may be attributed to the presence of some integrated H_3_O^+^ and water molecules. Directly above the electric double layer was a diffuse layer made up of anions: OH^-^, Cl^-^, H_2_PO_4_^-^, NaHPO_4_^-^, and HPO_4_^-2^, water, and cations. Within this layer, the Debye length extended only a few nanometers.

**Fig 9:**
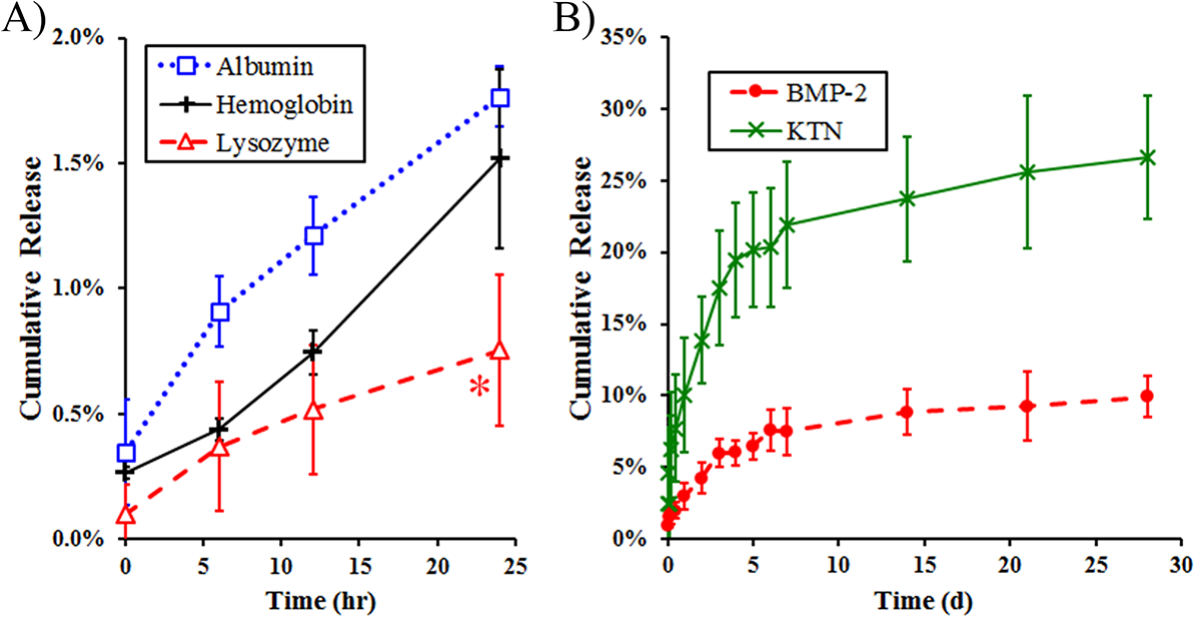
Rates of release of proteins out of bulk KTN gels. A) Globular proteins diffused out of gels in the following order: negatively-charged albumin, neutral hemoglobin, and positively-charged lysozyme. At the 24-hr time point, the amount of lysozyme released was significantly lower (*p < 0.01) compared to that of albumin and hemoglobin. B) BMP-2 had a slow release profile in PBS (pH 7.4) medium, suggesting tight electrostatic association with the KTN matrix and BMP-2 intermolecular aggregation due to the salting-out effect. KTN bulk degradation was faster relative to the BMP-2 release.

The non-covalent coulombic ability of KTN to sequester and release proteins was further analyzed via a KTN gel release assay. This involved the quantification of the release of globular proteins (with varying pI) from loaded KTN gels. Results (Fig 9A) indicated that electrostatic interactions dictated their release kinetics, even in medium with salts (50 mM KCl). At pH 7.4, the proteins have the following net charges: negative for keratin (pI = 5.3), negative for albumin (pI ~ 5), almost neutral for hemoglobin (pI ~ 7), positive for lysozyme (pI ~ 11), and positive for BMP-2 (pI = 9). Albumin was detected in the supernatant at the highest quantities at all time points (0, 6, 12, and 24 hr), which can be attributed to KTN-albumin repulsion, leading to faster diffusion out of KTN gels. A relatively slower release profile was observed in hemoglobin-loaded gels. The rate of diffusion is also dependent on the MW of the drug, with smaller molecules being released faster. Theoretically, lysozyme having the lowest MW (14 kDa versus the 66-kDa albumin and the 64-kDa hemoglobin) should diffuse out the quickest. However, due to the dominating electrostatic attraction between oppositely charged lysozyme and KTN, lysozyme diffused from the gels slowest. The negatively-charged KTN matrix has been shown by other research groups to absorb positively-charged substances with the rate of their diffusion regulated by charge effects.[18] Two-way ANOVA demonstrated significant differences between the type of loaded proteins (p = 0.0124), time (p < 0.0001), and interaction between the two variables (p = 0.048). At the 24-hr time point, a Bonferroni post-test showed that the lysozyme concentration in the supernatant was significantly lower (minimum of p < 0.01) than those of albumin and hemoglobin.

The moderately-strong electrostatic interactions between BMP-2 and the KTN matrix resulted in slow BMP-2 release kinetics in PBS at 37°C (Fig 9B). After 1 month *in vitro*, only 9.91 ± 1.43% (n = 3) of the available BMP-2 diffused out of the KTN gel. This rate of release was even slower than the bulk gel degradation and release of KTN proteins into the PBS medium, 26.64 ± 4.32% (n = 3) at the experimental endpoint. A 100 μg/mL = 2778 nM of BMP-2 was employed to make KTN-BMP-2 gels. At this concentration, BMP-2 was partly insoluble in PBS (salting-out effect). Hence, the observed slow BMP-2 release from KTN may likely be caused by both electrostatic binding to the gel scaffold and tight BMP-2 cohesion, which limited its water solvation and availability.

Previous studies in our group utilizing KOS as a carrier with similar initial BMP-2 concentration loading in PBS demonstrated that 80.16 ± 15.55% (n = 3) of BMP-2 was released at the 28-day time point.[11] Although it is anticipated that BMP-2 will bind more strongly with KOS than with KTN (because KOS has a lower pI, and is thus more negative), KOS gels degraded more quickly (75% degradation in 1 month), resulting in bulk release of the BMP-2.

The coulombic attraction between KTN and BMP-2 was fully lost when keratins were placed in a PBS liquid with pH of 4.5. In that environment, keratin switched its net charge from negative to positive while BMP-2 remained highly positive. SPR data demonstrated that even at increasing BMP-2 analyte concentrations, negligible association and dissociation signals were evident (Fig 8B), suggesting intermolecular repulsion and the absence of non-covalent interactions between KTN and BMP-2. In this acidic PBS (pH = 4.5), BMP-2 was soluble at all concentration levels used for SPR (up to 6944 nM = 250 μg/mL).

Lastly, it should be noted that BMP-2 growth factor proteins utilized in this study were originally diluted from a 1.5 mg/mL stock solution with pH of 4.5, which also contained the following excipients: 5 mg/mL sucrose, 25 mg/mL glycine, 3.7 mg/mL L-glutamic acid, 0.1 mg/mL NaCl, and 0.1 mg/mL polysorbate 80.[101] Hence, surface and bulk BMP-2 binding and release behavior may have been partially affected by possible interactions with these additives, especially with the charged amino acids: glycine and glutamic acid (pI of ~ 6 and ~ 3, respectively). Keratin, glycine, and glutamic acid all have an acidic pI, thus compete with each other to bind to positively-charged molecules. Accordingly, in the absence of negatively-charged excipients, the electrostatic attraction of KTN to BMP-2 may be greatly enhanced.

## Conclusions

Biocompatible, hair-derived keratin-based biomaterials, and particularly reductively produced kerateines (KTN), have the potential to become widely utilized drug-delivery vehicles that can be employed in a variety of tissue engineering applications. The free thiols of KTN can irreversibly link to gold substrates or form strong intermolecular covalent disulfide bonds to create longer-lasting gels. We have shown that binding and release of loaded model proteins in the negatively charged KTN network is mediated by electrostatic or coulombic interactions that are directly dependent on pH and salt concentration. Lower pH and higher salinity resulted in the formation of more stable KTN gels but also resulted in faster release of positively-charged molecules like BMP-2. KTN-to-BMP-2 association was found to be moderately-strong at neutral pH, even in the presence of physiological salts. Acidification of the salt medium caused KTN protonation, which led to the repulsion, non-interaction, and release of BMP-2.
